# Applicability of Downscaling Land Surface Temperature by Using Normalized Difference Sand Index

**DOI:** 10.1038/s41598-018-27905-0

**Published:** 2018-06-22

**Authors:** Xin Pan, Xi Zhu, Yingbao Yang, Chen Cao, Xize Zhang, Liangliang Shan

**Affiliations:** School of Earth Science and Engineering, Hohai University, 8 Buddha City West Road, Nanjing, 210098 China

## Abstract

Land surface temperature (LST) in coarse spatial resolution derived from thermal infrared satellite images has limited use in many remote sensing applications. In this study, we improve our previous approach (multiple remote-sensing index approach of random forest) to downscale LST derived from Landsat 8 and MODIS in an arid oasis - desert ecotone of Zhangye city by designing a normalized difference sand index (NDSI), by the removal of land cover datasets and by the input of SAVI, NDBI and NDWI to downscale LST. Our result demonstrates that NDSI can determine the characteristic of the desert region, and that the distribution of downscaled LST matches those of oasis-desert ecosystems. Relative to the ground observation of HiWATER, our approach also produces relatively satisfactory downscaling results at July 21 (2013), with R^2^ and root-mean-square error of 0.99 and 1.25 K, respectively. Compared with other methods, our approach demonstrates higher accuracy and minimization of the retrieved Landsat 8 LST in the desert region. Optimal availability occurs in the vegetation and desert region. Our approach is suitable to LST downscaling in all seasons, especially in spring and summer. The model can further be applied in middle-high and middle-low spatial resolutions. The usefulness of the model is relatively satisfactory in the humid region (Nanjing city) but less accurate in the arid region.

## Introduction

As an important parameter, the land surface temperature (LST) is always used for characterizing surface-energy balance and serves as the key factor in biophysical chemistry processes^1–5^. This parameter is extensively used in common applications, such as evapotranspiration and urban heat environment monitoring^6–10^. Satellite thermal infrared remote sensing (TIRS) can be used to derive LSTs. However, acquiring LSTs in a fine spatial resolution proves a challenge owing to the coarse spatial resolutions of TIRS.

LST downscaling provides an important method to increase the spatial resolution of relatively low-resolution LST images. In the most recent decades, to increase coarse resolution of LST, an amount of downscaling algorithms have been proposed. These models can be categorized as the model on the physical mechanism of downscaling and the statistical regression models^11–16^. The physical models, such as modulation-based methods, which established a function of LST or thermal radiation brightness and land cover types based on the principle of thermal radiation and spectral mixture analysis, have been proven to achieve an excellent downscaling effect^17–21^. Limited by the complexity of the physical models, the statistical models have become the most prevalent owing to their ease of use and acceptable downscaling accuracy. Two most common statistical models are the disaggregation procedure for radiometric surface temperature (DisTrad) method^22^ and the algorithm for sharpening thermal imagery (TsHARP)^23–25^. These two models present the linear or nonlinear relationships between LST and vegetation index. Although these models can be effectively employed to downscale LST over the vegetated areas, downscaling results are probably unsatisfactory in arid non-vegetated regions resulting from a lack of dynamic variation of vegetation index.

Recently, certain developments on LST downscaling in the arid non-vegetated regions have been improved by bringing in other predictors apart from the vegetation index, such as the indices of non-vegetated surfaces (soil, impervious surface, etc.) and surface reflectance^26–28^. One of these approaches is the recently proposed multiple remote sensing index approach of random forest (MIRF)^28^. MIRF brings various remote sensing indices into the random forest regression to downscale LST, and the downscaling performance is acceptable. Though some desert remote sensing indices were designed, they can not distinguish the sandy desert from the bail soil because of the similar reflectance differences of bands^29^. Limited by the lack of current remote sensing indices, which can characterize the sandy desert, the accuracy of downscaled LST in the arid non-vegetated region was less than that in the vegetated region.

Therefore, the current study proposes a new remote sensing index called a normalized-difference sand index (NDSI), which can characterize the sandy desert. Using that index, we further improve the original MIRF to downscale LST in the arid region. To distinguish the proposed approach from MIRF, we named the proposed approach as the approach of random forest using NDSI (NDSI-RF). By the visual and quantitative analyses, the downscaled LST are compared with LST obtained by the previous downscaling methods. The applicability of our method in different land covers as well as the applicability of our method in different seasons have been presented. Furthermore, the applicability of our method for different sensors as well as the applicability of our method in the humid region have been presented. The rest of this paper is organized as follows. Section 2 validates the downscaling results and analyses the applicability of our method. Section 3 discusses the improvement of MIRF. Section 4 demonstrates the design of a new remote sensing index and proposed method. Section 5 presents the study area and data. Section 6 concludes the paper.

## Results

### Downscaling Results

#### Spatial Distribution of Remote sensing indices and LST

**Figure 1:**
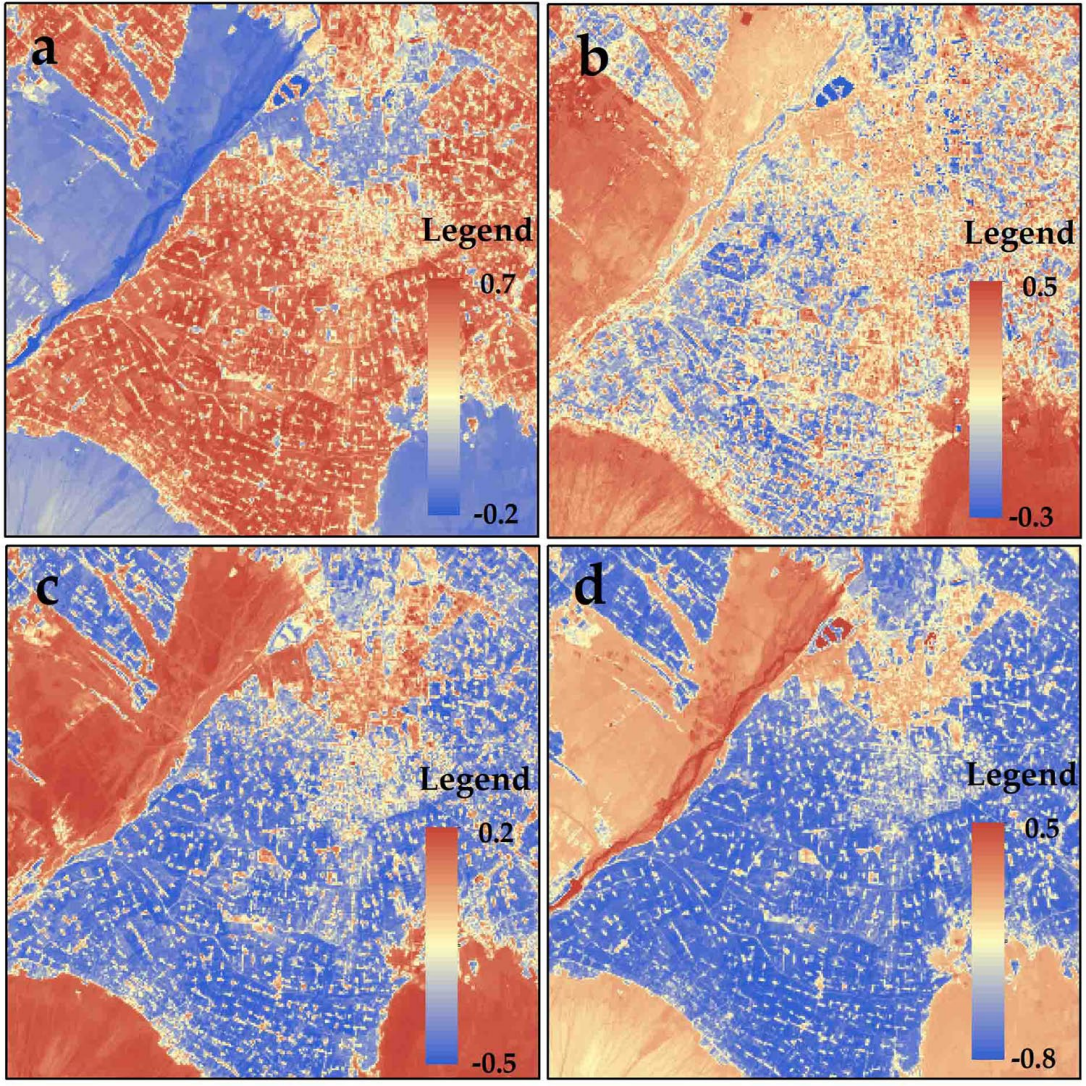
Distributions of remote sensing indices in Zhangye City: (**a**) SAVI, (**b**) NDSI, (**c**) NDBI, (**d**) NDWI.

The four remote sensing indices, namely, soil-adjusted vegetation index (SAVI), NDSI, normalized difference building index (NDBI), and normalized difference water index (NDWI), were extracted from the Landsat 8 Operational Land Imager (OLI) in the resolution of 90 m (Fig. 1). The oasis was located in the middle of the study area of Zhangye City with the luxuriant vegetation and a high SAVI of more than 0.5. In the desert located in the corner of study area (excepted for the northeast), a high NDSI (more than 0.5) occurred. In the urban area of the northern region, the medium remote sensing indices appeared. The water area (Heihe River) located at the boundary of desert and oasis in the west, had the largest NDWI. The distribution of remote sensing indices matched with the land cover.

**Figure 2:**
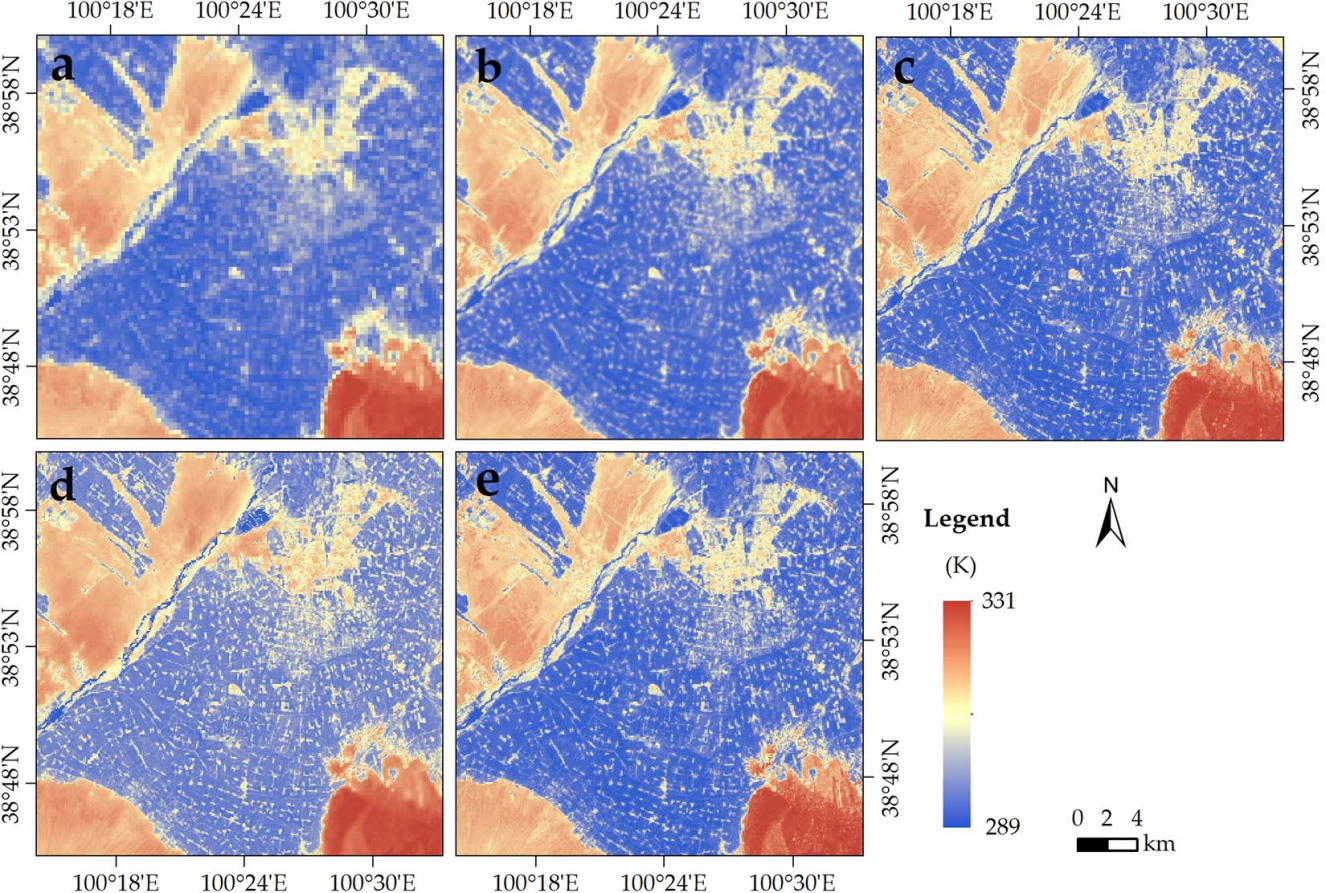
Distributions of (**a**) Landsat 8 retrieved LST (270 m), (**b**) Landsat 8 retrieved LST (90 m), (**c**) NDSI-RF LST (90 m), (**d**) Distrad LST (90 m), and (**e**) MIRF LST (90 m).

Figure 2a presents the distribution of LST (270-m retrieved Landsat 8 LST). The average value of LST was 307.6 K. The lowest LST (approximately 301.8 K) was detected in the middle oasis region with a high SAVI, whereas the highest LST (higher than 318.3 K) was detected in the desert region with high NDSI. There is a medium LST (nearly 309.0 K) in the urban region with medium remote sensing indices. Figure 2c presents the 90-m downscale LST using NDSI-RF. The average LST was 307.9 K. Its distribution is similar to the 270-m retrieved Landsat 8 LST. Similarly, the lowest, relatively low (309.8 K), and highest temperatures (318.6 K) were detected in the region of vegetation, building areas, and desert, with the value of 301.7 K, 309.8 K and 318.6 K, respectively. However, more detailed information were exited in the downscaled LST.

The LST distributions in the resolution of 270 m and 90 m were both obviously related to the remote sensing indices. The distribution of LST matched those of the remote sensing indices, and they all matched the oasis-desert ecosystems. The spatial distributions of the four remote sensing indices and the four types of land cover (vegetation, desert, impervious surface, and water) were consistent, and the four types of land cover can be characterized by these four these remote sensing indices.

#### Evaluation of downscaled LST

**Figure 3:**
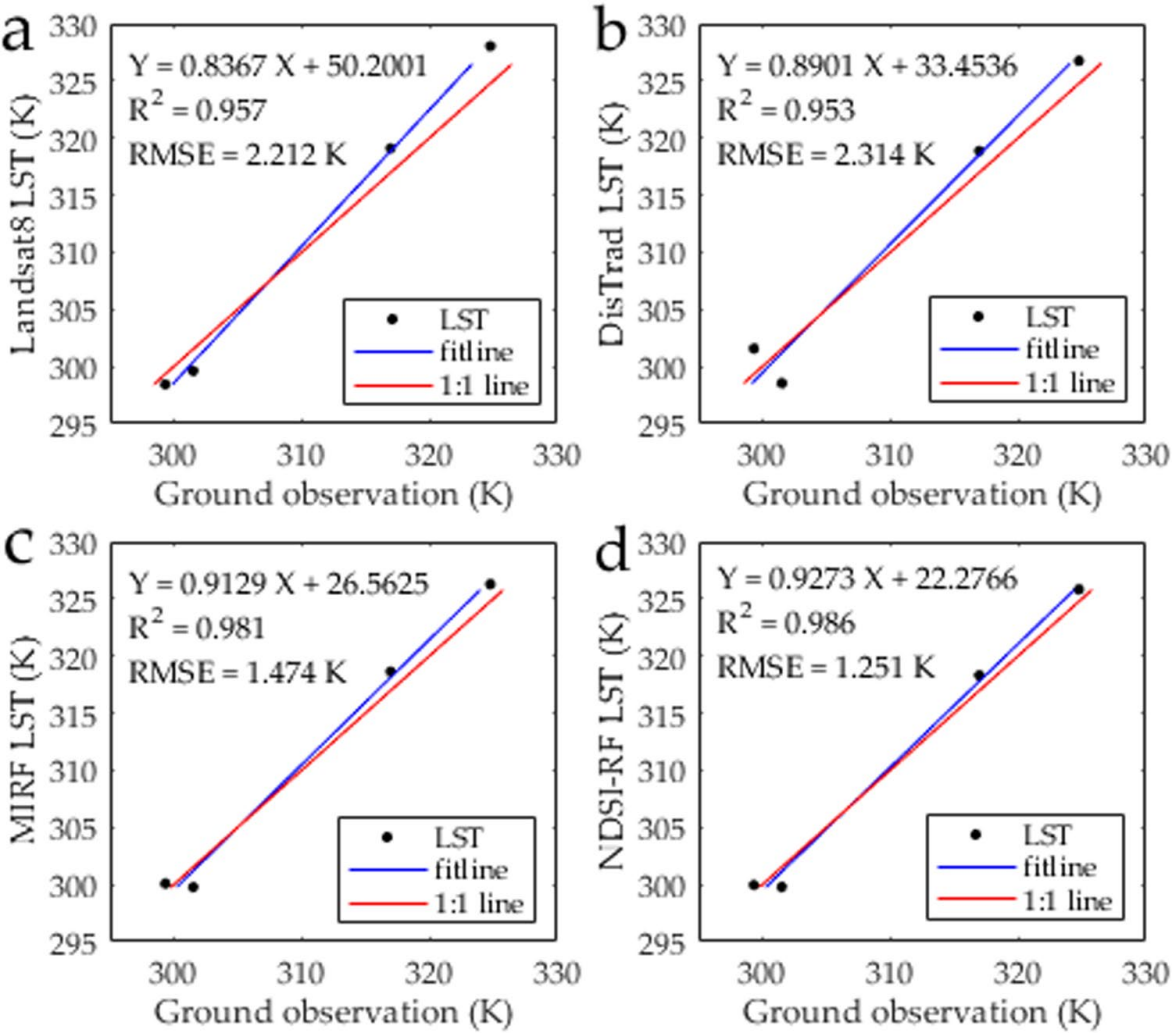
Direct validation of (**a**) Landsat 8 retrieved LST (90 m), (**b**) DisTrad LST (90 m), (**c**) MIRF LST (90 m), and (**d**) NDSI-RF LST (90 m) at all four sites.

Figure 3 illustrates the relationship of ground observations and remote sensing/downscaled LST at all four sites in the satellite overpassing moment. Relative to the observation in 10 minutes, the downscaled LST using NDSI-RF was generally accurate, with root-mean-square error (RMSE) and coefficient of determination (R^2^) values of 1.25 K and 0.99, respectively. Obviously, its accuracy overwhelmed the accuracy of Landsat 8 retrieved LST, which has RMSE and R^2^ values of 2.21 K and 0.96. Table 1 also shows the bias of remote sensing/downscaled LST and ground observations at all four sites. There is a relatively good agreement of the NDSI-RF downscaled LST and ground observations at most of sites, with bias of −1.74–1.33 K. Compared with the severe underestimation of Landsat 8 retrieved LST (2.12–3.26 K) at Gobi and desert sites, NDSI-RF obviously improved the accuracy of LST at all sites (1.06–1.33 K).

**Table 1:** Bias of (a) Landsat 8 retrieved LST, (b) DisTrad LST, (c) MIRF LST, and (d) NDSI-RF LST at four sites.

Site	Landsat 8 (K)	DisTrad (K)	MIRF (K)	NDSI-RF (K)
Wetland	−0.90	2.24	0.76	0.62
Maize	−1.91	−2.97	−1.76	−1.74
Gobi	2.12	1.89	1.66	1.33
Desert	3.26	2.00	1.51	1.06

**Figure 4:**
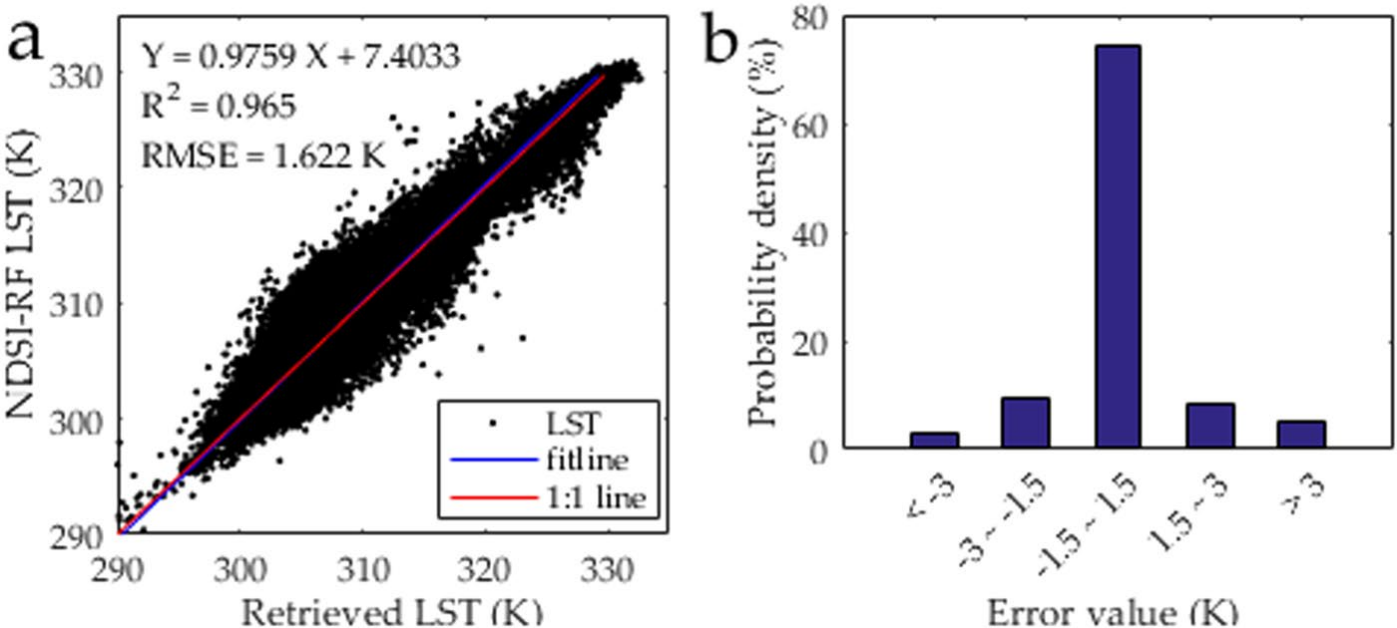
Comparison of the 90-m NDSI-RF downscaled and 90-m Landsat 8 retrieved LST in Zhangye City: (**a**) Scatter plot, (**b**) Error probability.

**Figure 5:**
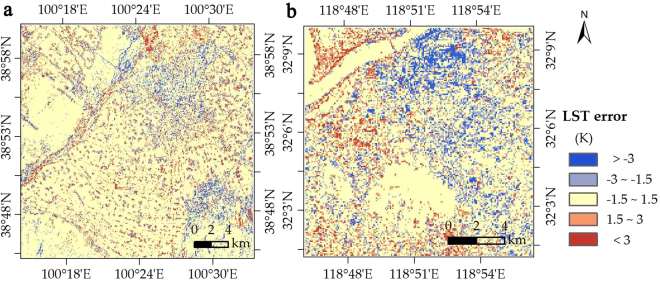
Spatial distribution of the discrepancy of the 90-m NDSI-RF downscaled and 90-m Landsat 8 retrieved LST in (**a**) Zhangye City and (**b**) Nanjing City.

Compared with the retrieved 90-m Landsat 8 LST, the 90-m downscaled LST had pixel-average R^2^ and RMSE values of 0.97 and 1.62 K for the entire image, respectively (Fig. 4a). The amount of pixels with LST errors of −1.5 to 1.5 K, −3.0 to −1.5 K, 1.5 to 3.0 K, <−3.0 K, and ≥3.0 K, accounted for 74%, 9%, 8%, 3%, and 6% of all the pixels, respectively (Fig. 4b). The 90-m downscaled LST proved to be reliable (with a bias of less than 1.5 K) in approximately three quarters of the study area. The obvious underestimation occurred in the northern urban area, whereas the overestimation occurred in the west along the Heihe River (Fig. 5a). Therefore, the direct and cross validation of downscaled LST both demonstrated the availability of the proposed approach.

#### Comparison of Approaches

In Fig. 2, the 270-m Landsat 8 retrieved LST image was downscaled by three approach, including DisTrad, MIRF and NDSI-RF approach. More detailed information were detected in the downscaling result of these three approach, especially in the oasis and desert region (Fig. 2c–e). Compared with the ground observations, the R^2^ (RMSE) of DisTrad, MIRF, and NDSI-RF approach are 0.95, 0.98, and 0.99 (2.31 K, 1.47 K, and 1.25 K), respectively (Fig. 3). In detail, the bias values of DisTrad, MIRF, and NDSI-RF approach are −2.97 −2.24 K, −1.76 −1.66 K, and −1.74 −1.33 K, respectively (Table 1). Notably, the bias of LST obtained using NDSI-RF approach at these two sites are 1.06 K and 1.33 K, whereas the bias of LST downscaled by DisRrad (MIRF) are up to 1.89 K and 2.00 K (1.66 K and 1.51 K). The performance of NDSI-RF approach was better than that of the DisTrad or MIRF approach, especially at the Gobi and desert sites in the desert region.

### Applicability of NDSI_RF

#### Applicability in different land covers

**Figure 6:**
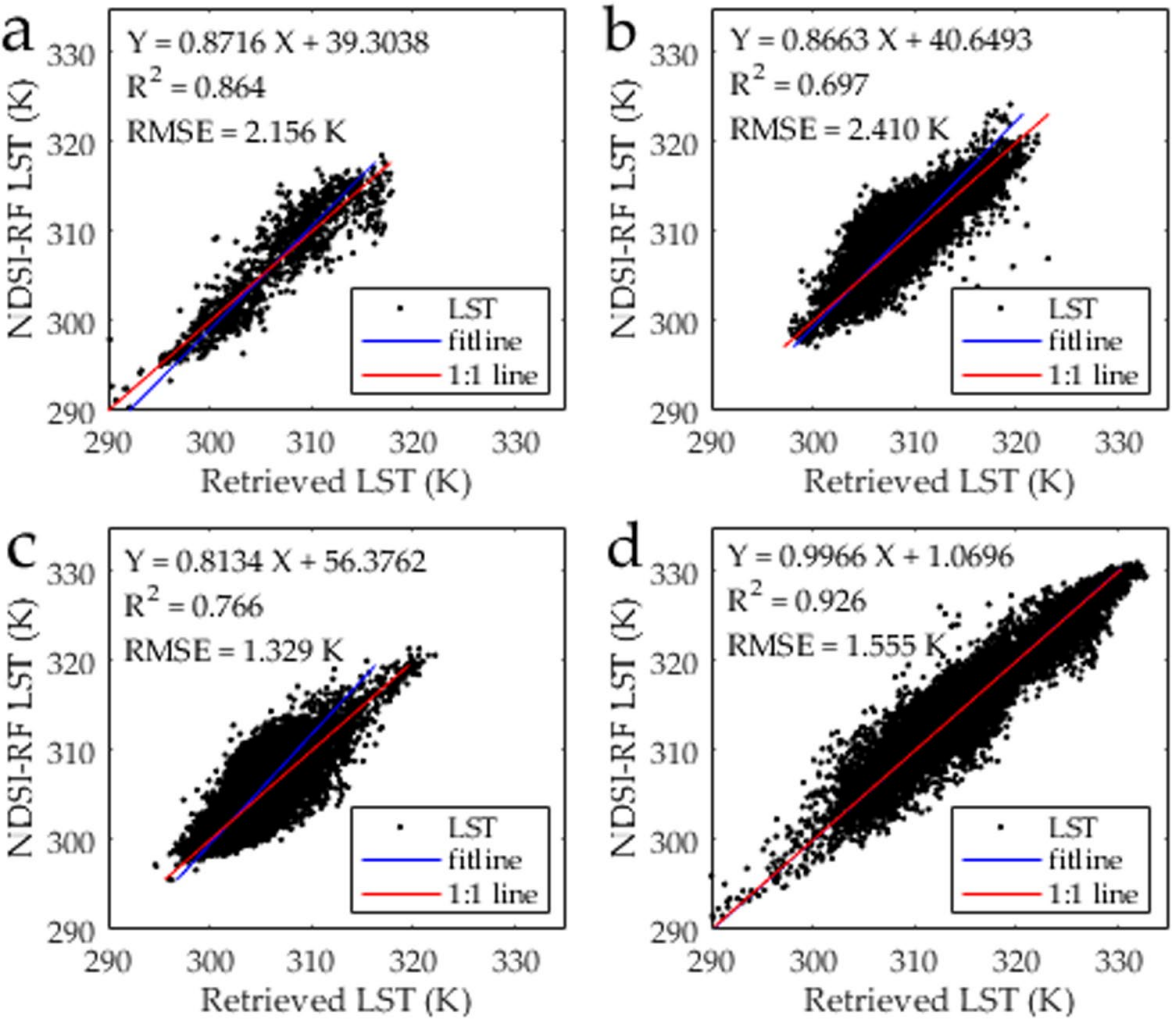
Comparison of the 90-m NDSI-RF downscaled and 90-m Landsat 8 retrieved LST in the following regions: (**a**) water, (**b**) building, (**c**) vegetation, and (**d**) sand.

In the study area of the arid region, four main land covers are identified, namely, vegetation, desert, building, and water. To demonstrate the applicability of our approach in different land covers, the downscaled LST were evaluated by the 90-m Landsat 8 retrieved LST in the region with four different land covers (Fig. 6). The RMSE (R^2^) of downscaled results in the area of water, vegetation, impervious surface, and sand were 2.16 K (0.86), 1.33 K (0.77), 2.41 K (0.70), and 1.56 K (0.93), respectively. Obviously, the downscaled LST is in a good accordance with the 90-m retrieved LST in the desert region with a relatively satisfactory accuracy. The downscaled LST also had a good accuracy in the region of vegetation with the RMSE of 1.33 K. The lowest accordance of downscaled and retrieved LST (R^2^: 0.70) occurred in the region of building with the largest RMSE of 2.41 K for downscaled LST. Similarly, the downscaling performance is less satisfactory in the region of water with the RMSE of 2.16 K. Therefore, our approach displays its capacity of downscaling in the region of desert and vegetation. In the water and impervious surface areas, the downscaling performance is relatively unsatisfactory. Considering that our study mainly aims to improve the downscaled LST in the desert region and the region of vegetation and desert dominating the arid region, our approach demonstrates its advantage in the LST downscaling in the arid region.

#### Applicability in different seasons

**Figure 7:**
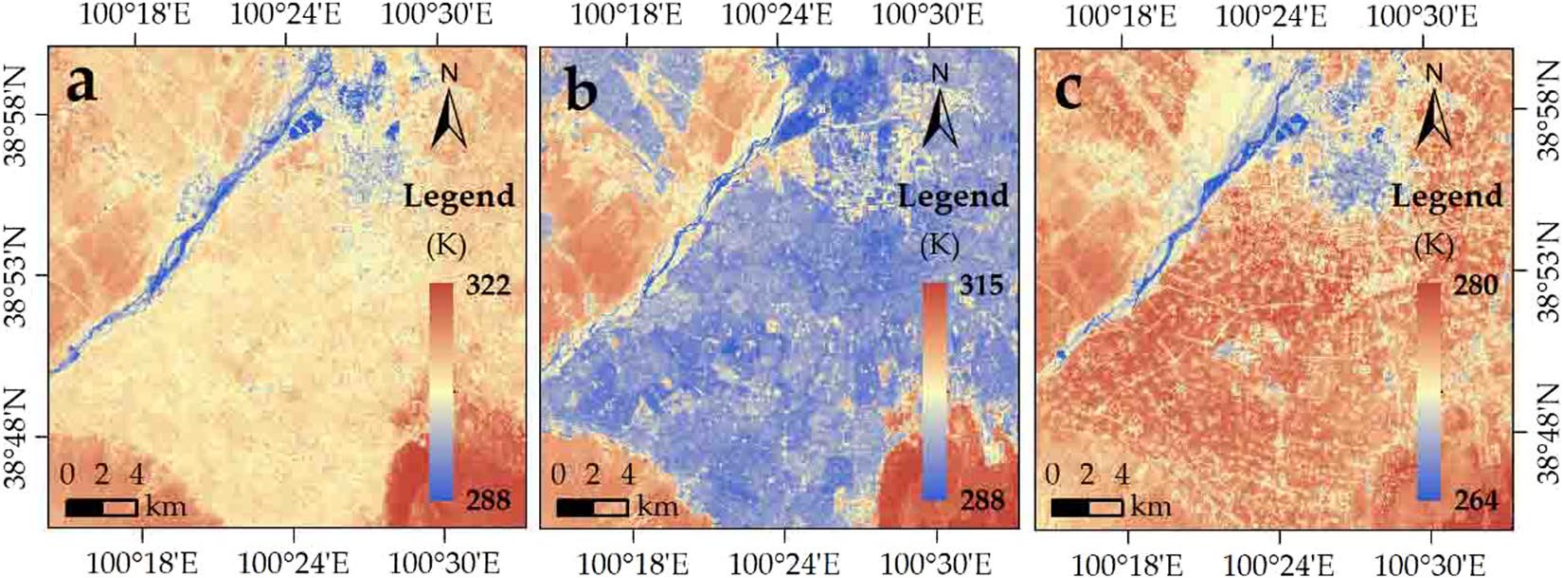
90-m Landsat 8 downscaled LST in (**a**) spring (16 April 2013), (**b**) autumn (9 October 2013), and (**c**) winter (28 December 2013).

Similar to the downscaling in the summer (Fig. 2c), Fig. 7 presents the downscaling results of our approach in the other three seasons. Evidently, the accuracy of NDSI-RF downscaling results were higher than the accuracy of the Landsat 8 retrieved LST in all four seasons (Tables 1 and 2). Compared with the ground observations, a bias of the 90-m NDSI-RF LST exited −1.17 to 0.24 K, −3.86 to 0.65 K, and −2.43 to −1.03 K at four sites in the spring, autumn, and winter, respectively. Therefore, the better applicability of NDSI-RF appeared in the spring and summer.

**Table 2:** Bias of 90-m Landsat 8 downscaled LST and NSDI-RF downscaled LST at four sites in spring (16 April 2013), autumn (9 October 2013) and winter (28 December 2013).

Site	2013.4.16		2013.10.9		2013.12.28	
	Landsat8 (K)	NDSI-RF (K)	Landsat8 (K)	NDSI-RF (K)	Landsat8 (K)	NDSI-RF (K)
Wetland	−3.87	−1.17	−3.16	−1.84	−3.01	−2.43
Maize	0.61	0.03	−4.57	−3.89	−2.14	−2.03
Gobi	−0.87	−0.80	0.30	−0.09	−1.56	−1.45
Desert	0.95	0.24	1.24	0.65	−1.53	−1.03

In detail, the NDSI-RF downscaled LST at Gobi and desert sites (in the desert region) had a relatively steady accuracy in all seasons, whereas that in the oasis region (wetland and maize sites) varied with the seasons. The downscaled LST had a relatively high accuracy in the spring and summer but decreased in autumn and winter, which may be relative to the spare vegetation in the oasis after harvest.

Generally, the NDSI-RF approach can be used to downscale LST in all seasons, especially in spring and summer. The best availability also detected in the desert regions in the autumn and winter.

#### Applicability for Satellite Images in Middle-Low Resolution

**Figure 8:**
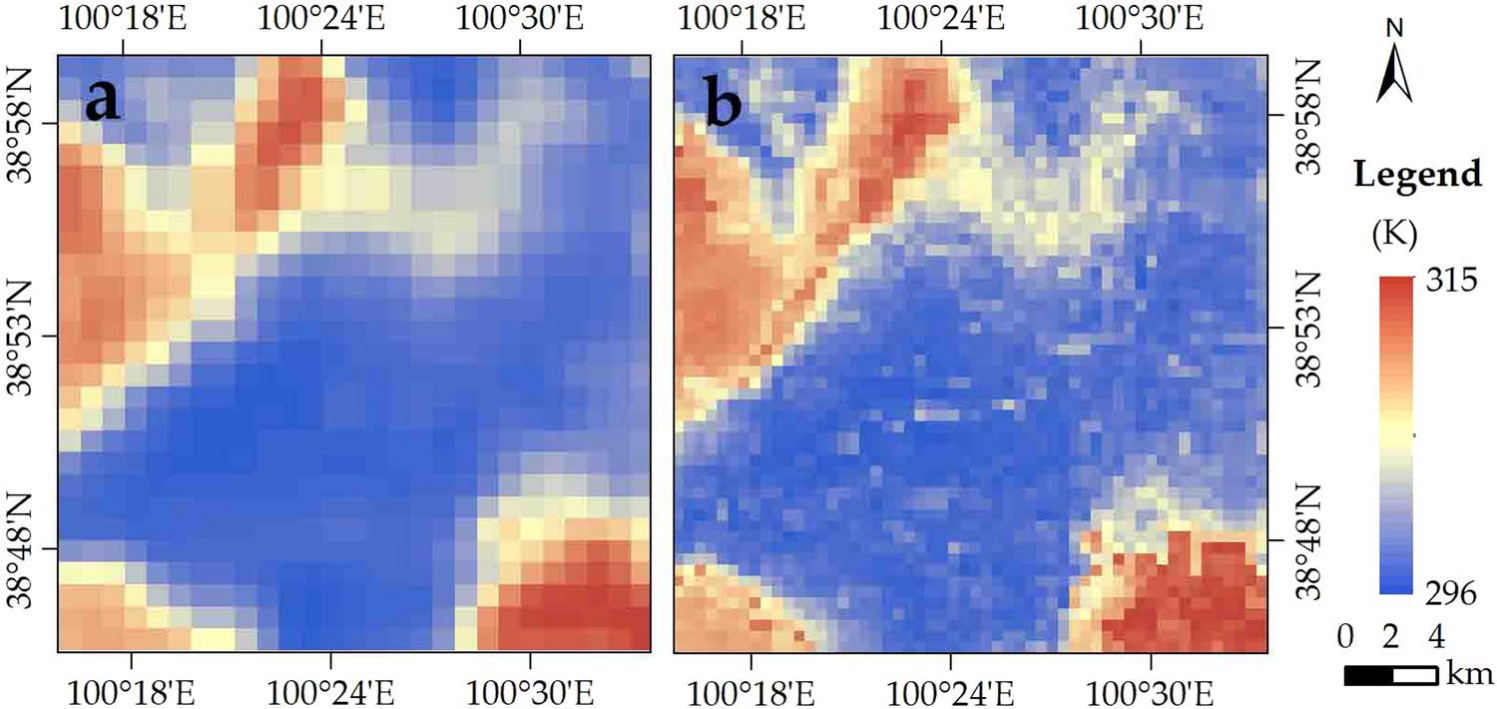
Distribution of (**a**) MOD11 (1 km) and (**b**) NDSI-RF downscaled LST (500 m) in Zhangye City on 3 September 2012.

Figure 8 illustrates the 1-km LST distribution of MOD11 which is the LST product of Moderate Resolution Imaging Spectroradiometry (MODIS) and 500-m downscaled LST. More detailed LST information were detected after downscaling, especially in the urban area and the desert. The average LST was 302.10 K and 302.17 K for the MOD11 and downscaled LST, respectively.

Relative to the ground observations at all four sites, the errors of NDSI-RF LST were −0.48 to 3.33 K, whereas the error of MOD11 LST were −9.17 to 1.80 K (Table 3). There is a decrease of the LST error for downscaling at almost all sites (expect for the wetland site), especially at the Gobi and desert sites. Relative to the MOD11 LST, the error decreased from over −5 K to near 0 K at these two sites. Therefore, our approach proves its applicability in the desert region for MODIS, which is one of most representative LST products in a middle-low resolution.

**Table 3:** Bias of MOD11 LST (1 km), and downscaled LST (500 m).

Site	MOD11 1 km (K)	NDSI-RF 500 m (K)
Wetland	1.80	3.33
Maize	−0.69	0.29
Gobi	−5.22	−0.16
Desert	−9.71	−0.48

#### Applicability in the humid region

**Figure 9:**
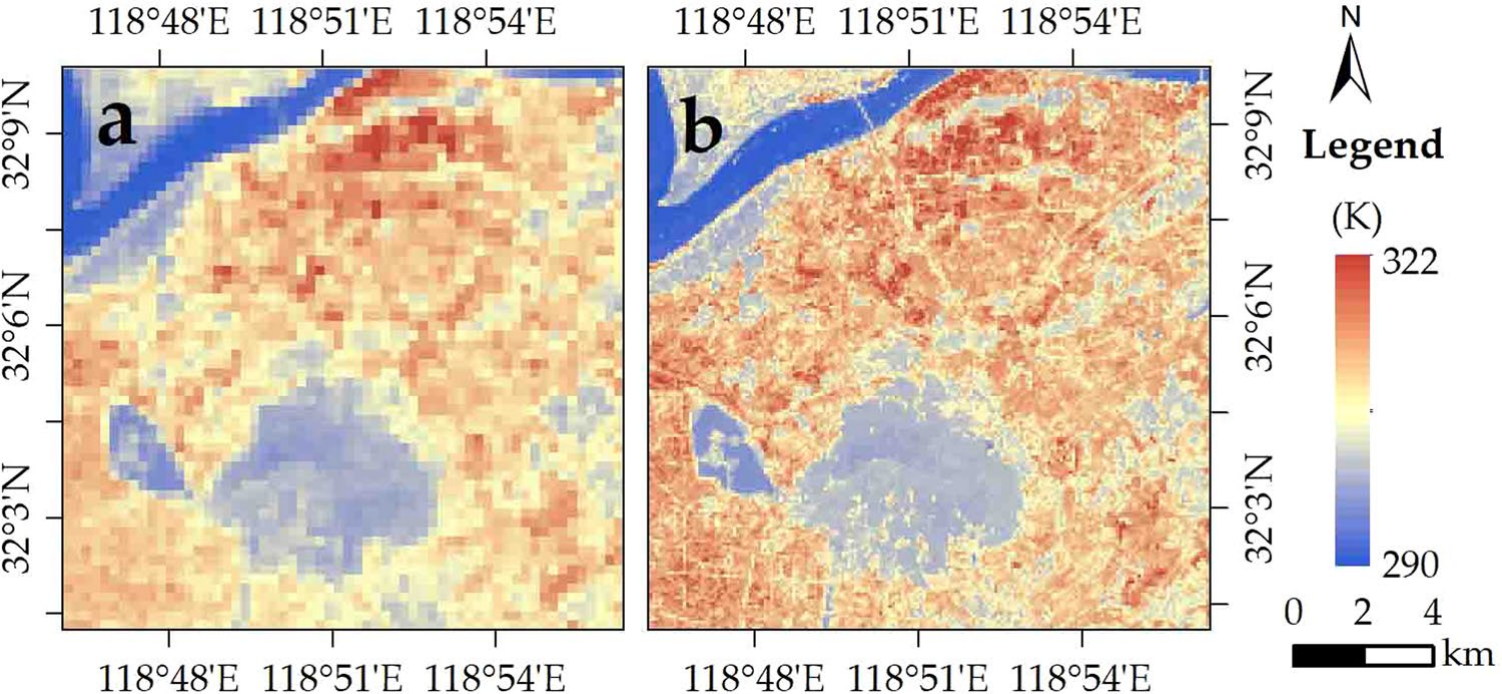
Distribution of (**a**) Landsat 8 (270 km) and (**b**) NDSI-RF downscaled LST (90 m) in Nanjing City.

**Figure 10:**
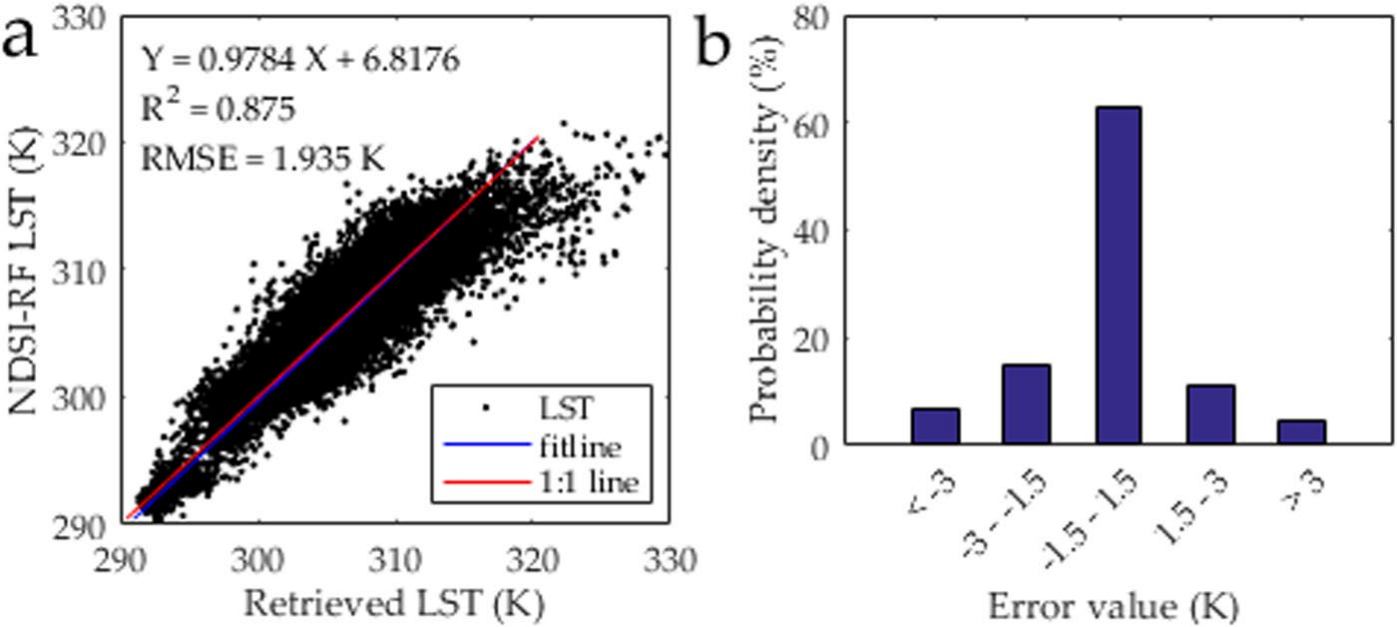
Distribution of (**a**) Landsat 8 (270 km) and (**b**) NDSI-RF downscaled LST (90 m) in Nanjing City.

On August 11 in 2013 in Nanjing, the 270-m Landsat 8 LST was downscaled to 90-m LST (Fig. 9). In the comparison with the 90-m Landsat 8 retrieved LST, there is a relatively satisfactory accuracy for the 90-m downscaled LST, with pixel-average R^2^ and RMSE of 0.88 and 1.97 K, respectively (Fig. 10a). The amount of pixels with LST errors of −1.5–1.5 K, −3.0–1.5 K, 1.5–3.0 K, <−3.0 K, and ≥3.0 K accounted for 63%, 15%, 11%, 7%, and 4% of the entire image, respectively (Fig. 10b). In two thirds of pixels, the difference of the retrieved and downscaled LSTs was less than 1.5 K which is better than the accuracy of LST retrieval^30^. Thus, in most of the study area, the downscaling results were reliable (Fig. 5b). In the northern industrial area, there is a systemic overestimation. In addition, some pixels with underestimation were found in the northwest, specifically in the locality near the shores of the Yangzi River.

Compared with the ground observation (308.17 K), the retrieved and downscaled LSTs were 307.89 K and 308.26 K, respectively. The error of downscaled LST was 0.09 K, which was within the accuracy of LST retrieval. Therefore, the direct validation also confirms the availability of our approach.

In summary, the 90-m downscaled LST proved to be reliable (with a bias of less than 1.5 K) in approximately two-thirds of the area, except for the shores of the Yangzi River and the industrial zone in the northeast region. Compared with the cross validation of downscaled LST in the arid region (R^2^ and RMSE values of 0.97 and 1.62 K), although our approach proves its applicability in the humid region, its performance in the humid region is not as satisfactory as that in the arid region.

## Discussion

Obtaining the LST mapping in high resolution is necessary in a lot of applications, such as in monitoring ground water cycle and analyzing the urban heat island^1,31,32^. To the best of our knowledge, LST downscaling is an effective method to improve the spatial resolution of LST. Previous study proposed the RF approach using multiple relevant remote sensing indices to downscale the LST maps in the arid regions^28^. The downscaled LSTs obtained by the MIRF algorithm were evaluated in the arid region. Among these remote sensing indices, NDDI was chosen to characterize the desert. Although NDDI can recognize the desert from oasis, it cannot recognize the desert from the bail soil. Accordingly, MIRF algorithm limitedly improves the accuracy of downscaled LST in the desert region. Thus, the present study primarily designs a remote sensing index, which can characterize the desert and improve the MIRF algorithm in the arid region. NDSI-RF approach further improves both spatial resolution and accuracy of LST, especially in the arid desert regions. Different from MIRF approach, the land cover datasets are not necessary in our approach. Accordingly, the simplicity of downscaling improves and the accuracy of land cover have no influence on the downscaling result.

The applicability of the MIRF algorithm was discussed rudimentarily in the arid region in the previous study^28^. With an aim to improve the range of application, the applicability of the proposed approach has been comprehensively presented in our study, including the applicability in the region of different land covers, those in the different seasons, those for different sensors, and that in the moist region. Our approach is available to the LST downscaling in all seasons and is also worded well in the different climatic regions, especially in the arid desert region. The LST derived from TIRS images in the middle-high and middle-low resolutions can also be downscaled by our approach. The extensive applicability strongly supports the effectiveness of our approach.

Although NDSI-RF has been applied in downscaling for the sensors in the middle-high and middle-low resolutions, the combination of geostationary meteorological satellite images and polar satellite images perhaps helps simultaneously improve the spatial and temporal resolutions of downscaled LST^33,34^. Thus, the LST downscaling of multi-sensors to monitor the regional thermal environments using our approach requires further research in the future.

## Methods

### Normalized Difference Sand Index

**Figure 11:**
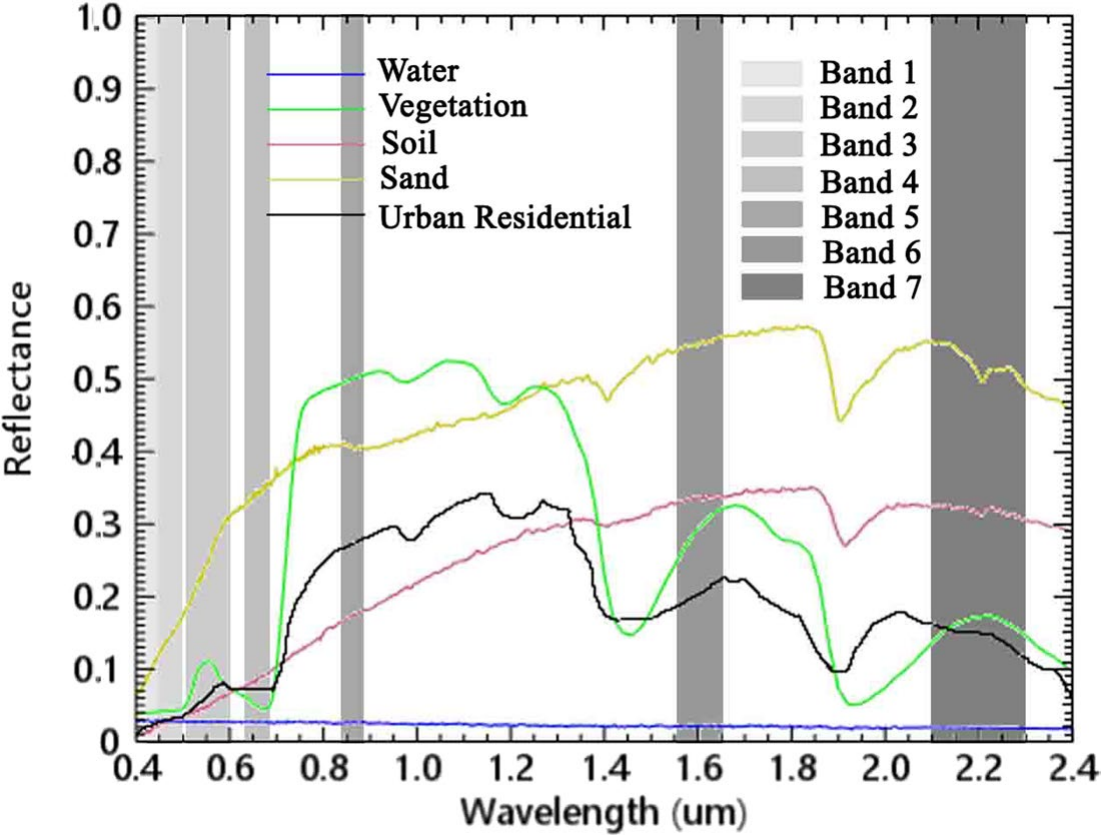
Reflectance of water, vegetation, soil and urban residential in the 0.4–2.5 μm spectrum.

For Landsat 8 OLI, seven solar reflectance bands (Bands 1–7) from 0.43–2.29 μm are identified. In the Advanced Spaceborne Thermal Emission and Reflection Radiometer spectrum library, there are hundreds of spectral signatures for materials which can identify the material^35^. The spectral signatures of sand, grass, soil, urban residential, and water, and the locations of Landsat 8 OLI solar reflectance bands (0.43–2.29 μm) were shown in Fig. 11.

Clearly, there is an increase of the reflectance of sand when the wavelength varies from 0.43 to 2.29 µm with a minimum in Landsat 8 OLI Band 1 (0.43–0.45 µm). Obviously, the reflectance values of others are also minimum in Band 1. Furthermore, in the wavelength of visible spectrum (0.4–0.76 µm), the reflectance of sand can be up to nearly 0.4 (in Band 4, 0.64–0.67 µm). Whereas those of others are lower than 0.1.

The spectral characteristic of sand suggests that the reflectance signals can be obtained using the difference between Band 4 signal, which is high, and Band 1 signal, which is significantly low. This difference distinguishes rather well between sand and other ground features. Considering this strong discrimination possibility, we propose NDSI to detect sand. The NDSI can be written as

1 $$NDSI=({\rho }_{band4}-{\rho }_{band1})/({\rho }_{band4}+{\rho }_{band1}),$$

where ρ_band1_ and ρ_band4_ are the reflectances of Bands 1 and 4 for Landsat 8 OLI, respectively. Obviously, the higher NDSI, the higher the content of sand dominates in a region.

The difference of the reflectance of Bands 1 and 4 for sand is evidently larger than that for soil, whereas the difference of the reflectance of Bands 7 and 4 for sand is similar to that for soil. Therefore, relative to other sand indices designed by other researchers, our index can distinguish the sand from soil better, and it is more suitable for the images of Landsat 8^29^.

### Downscaling Methods

Downscaling models between ancillary environmental predictors in a high resolution and LST in a coarse resolution have been established to enhance LST resolution. In previous research of LST downscaling with RF, we have proposed MIRF^28^. A few remote sensing indices related to land status were chosen; these factors include SAVI^36^, normalized multi-band drought index (NMDI)^36^, modified normalized difference water index (MNDWI)^37^, NDBI^38^, and normalized difference dust index (NDDI)^39^. However, NDDI is not helpful in distinguishing sand from bail soil, and it cannot also present the characteristic of sand in the desert region. Accordingly, the accuracy of downscaled LST is limited. Moreover, the accuracy of land cover datasets is important to the downscale performance. In the area of mixed land cover, MIRF fails in the accurate LST downscaling. Therefore, to improve MIRF, the re-selection of remote sensing indices and the removal of land cover datasets are necessary.

The current study selected four remote sensing indices related to the main ground features (vegetation cover, water cover, impervious surface cover, and desert) in the arid region, including SAVI, NDWI, NDBI and NDSI. These remote sensing indices and LST image with coarse resolution are regressed into the RF regression trees as follows:

2 $$LS{T}_{F}=f(SAV{I}_{C},NDW{I}_{C},NDB{I}_{C},NDS{I}_{C}),$$

where *SAVI*_*C*_, *NDWI*_*C*_, *NDBI*_*C*_, *NDSI*_*C*_, and *LST*_*F*_ are the SAVI, NDWI, NDBI, NDSI, and fitted LST in a coarse resolution, respectively.

The residual temperature (*e*) is defined as Eq. 2:

3 $$e=LS{T}_{F}-LS{T}_{{\rm{O}}}.$$

where *LST*_*O*_ is the original LST which the retrieved LST in our study.

Therefore, from the coarse-resolution LST, the simulated LST in a coarse resolution (*LST*_*C*_) can be derived as

4 $$LS{T}_{C}=f(SAV{I}_{C},NDW{I}_{C},NDB{I}_{C},NDSI{}_{C})+e.$$

Then, the above relationships were applied to the remote sensing indices in a high resolution. The downscaled LST (*LST*_*H*_) is acquired,

5 $$LS{T}_{H}=f(SAV{I}_{H},NDW{I}_{H},NDB{I}_{H},NDSI{}_{H})+e,$$

where *SAVI*_*H*_, *NDWI*_*H*_, *NDBI*_*H*_, and *NDSI*_*H*_ are the SAVI, NDWI, NDBI, and NDSI in a high resolutions.

Different from MIRF, we notably did not build the model for each land cover in our approach. The land cover datasets are irrelevant to the proposed model. The proposed approach is NDSI-RF approach.

### Evaluation Measures

Three measures were selected to evaluate the downscaling LST, including R^2^, bias, and RMSE. R^2^ is the coefficient of determination between the original and downscaled images. A high R^2^ indicates a satisfactory downscaling. This coefficient is shown as

6 $${R}^{2}=1-\frac{\sum {(LS{T}_{S}-LS{T}_{R})}^{2}}{\sum {(LS{T}_{S}-\overline{LS{T}_{R}})}^{2}},$$

where *LST*_*S*_ is the downscaled LST (Eq. [5]), LSTR is the reference LST, and $\overline{LS{T}_{R}}$ is the average of *LST*_*R*_. Bias and RMSE were used to test the errors between the retrieved and downscaled *LSTs*. Their definition are listed here

7 $$bias=\frac{{\sum }_{i=1}^{n}LS{T}_{S}-LS{T}_{R}}{n}$$

8 $$RMSE=\sqrt{\frac{1}{{\rm{n}}}\sum _{i=1}^{n}{(LS{T}_{S}-LS{T}_{R})}^{2}}$$

where *n* represent the amount of pixels.

### Data availability

The datasets generated during and/or analysed during the current study are available from the corresponding author on reasonable request.

## Study Area and Data

### Study Area

**Figure 12:**
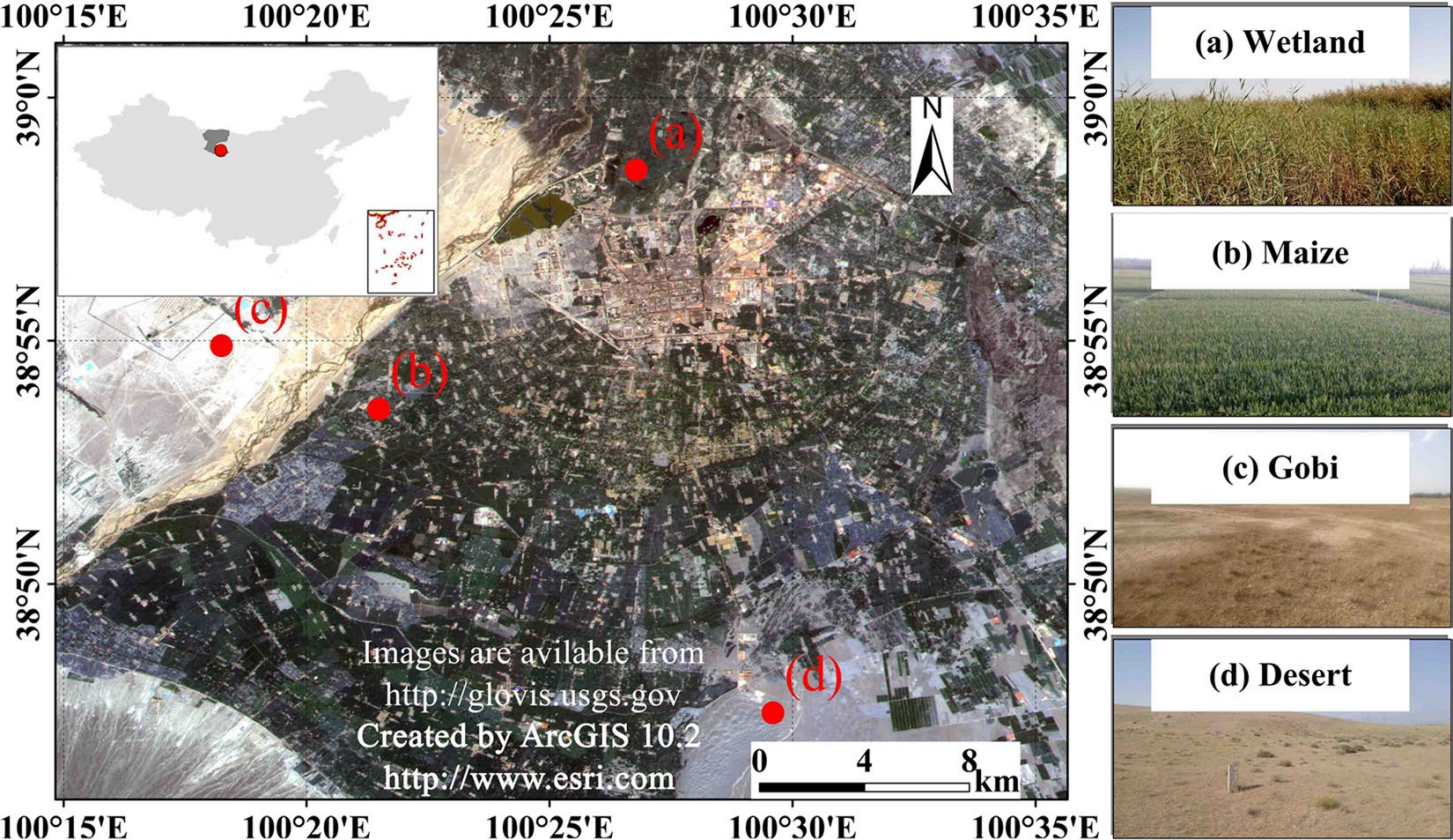
Sites and underlying surfaces of the study in Zhangye City (satellite images derived from United States Geological Survey).

The study areas are located in a part of Zhangye City (the typical arid region) and a part of Nanjing City (the typical humid region). Our study area in the arid region is located at an oasis–desert ecotone of Zhangye City (31°14′–32°37′N, 118°22′–119°14′E). In this area, an arid continental climate dominates. The months of October to May of next year are dry and the rainy periods are from June to September. The annual mean values of air temperature, precipitation and evaporation are 281 K, 1156 mm, and 2107 mm, respectively^40^. There are four main land cover types in the study area, including vegetation, desert, impervious surfaces and wetland. We choose four ground sites to represent the four land cover types, including wetland, maize, Gobi, and desert sites (Fig. 12). Wetland and maize sites are located in the oasis region, whereas Gobi and desert sites are located in the desert region. These sites belongs to the Heihe Watershed Allied Telemetry Experimental Research (HiWATER)^41^, and all ground observation data were provided by the Cold and Arid Regions Science Data Center at Lanzhou (WestDC)^42^. The land cover of the surface around the ground sites is quite homogeneous according to the field visit.

**Figure 13:**
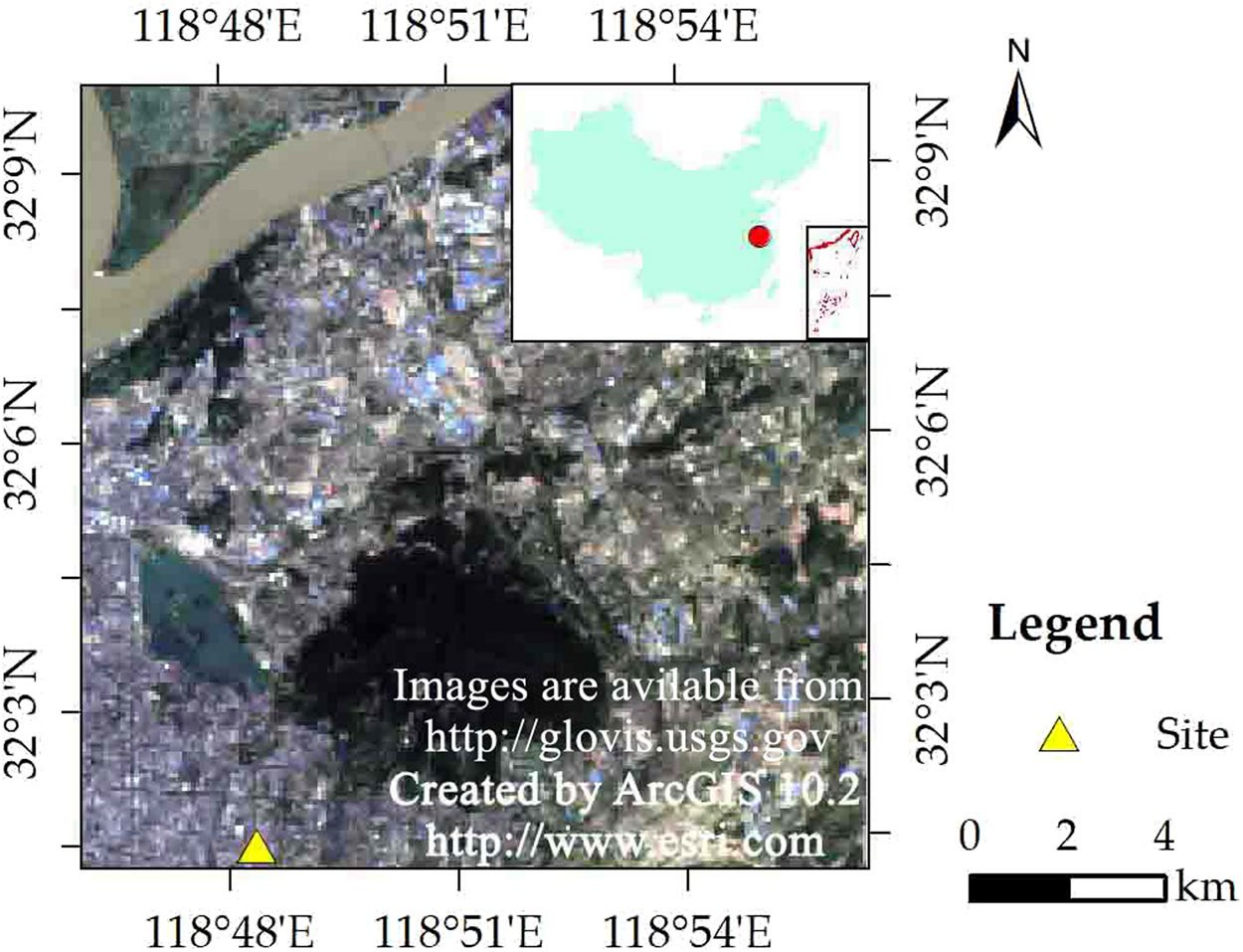
Site and underlying surfaces of the study in Nanjing City (satellite images derived from United States Geological Survey).

Nanjing (31°14″–32°37″N, 118°22″–119°14″E) is a metropolis in the southeastern of China. Influenced by the East Asian monsoon, a humid subtropical climate dominates in our study area. The annual average precipitation is 979 mm, and the annual mean air temperature is 289 K. The hottest and most humid period focused on the summer (from June to August)^43^. The study area in the humid region is a part of Nanjing City (Fig. 13). The area is characterized by four main land cover types, including impervious surface, vegetation, bare soil, and water. One ground site of the China Meteorological Administration was chosen to evaluate the LST downscaling.

### Data Description

The Landsat 8 OLI and TIRS images were chosen in this study and were provided by the United States Geological Survey in the 30 m and 100 m spatial resolutions, respectively (Table 4)^44^. The image of Zhangye City was acquired on July 21 (2013) to evaluate the LST downing during the summer in the arid region. The other images were also acquired on April 28, October 9 and December 28 in 2013 to determine the availability of our approach during the other seasons in the arid region. Moreover, an image of Nanjing was acquired on August 11 in 2013 to present the applicability of our approach in the humid region.

**Table 4:** Information of the selected datasets.

Datasets	Sources	Parameters	Temporal and Spatial Resolutions	Usages
Landsat 8	USGS	Surface reflectances	16 day, 100 m (TIRS)/30 m (OLI)	Downscaling
MOD11_L2	NASA MODIS	LST	1 day, 1 km	Applicability Analysis
MOD09GA		Surface reflectances	1 day, 500 m	Applicability Analysis
Land Cover	HiWATER	Land covers	1month, 30 m	Applicability Analysis
Site Observation		LST	10 min, meters	Validation

First of all, the Fast Line-of-sight Atmospheric Analysis of Hypercubes (FLAASH) atmospheric correction algorithm was used to adjust the OLI images^45^. For convenience, the 30-m OLI and 100-m TIRS images were both resampled into 90-m images. Furthermore, the 90 m OLI and TIRS images were also resampled by aggregation into 270-m images. Then, 90-m and 270-m LST can be retrieved from TIRS by generalized single-channel algorithm^46^. 270-m LST was downscaled by 90-m remote sensing indices derived by 90-m OLI images, and 90-m LST was used to evaluate the 90-m downscaled LST.

To present the applicability of our approach for the LST product in the middle-low resolution, MODIS data was chosen in September 3 (2012). The products were provided by the Level 1 and Atmosphere Archive and Distribution System. In this study, we used the land surface emissivity and LST obtained from the MOD11 in 1-km resolution, and the band reflectances obtained from the MOD09 in 500-m resolution to downscale the resolution of MOD11 LST from 1 km to 500 m.

**Figure 14:**
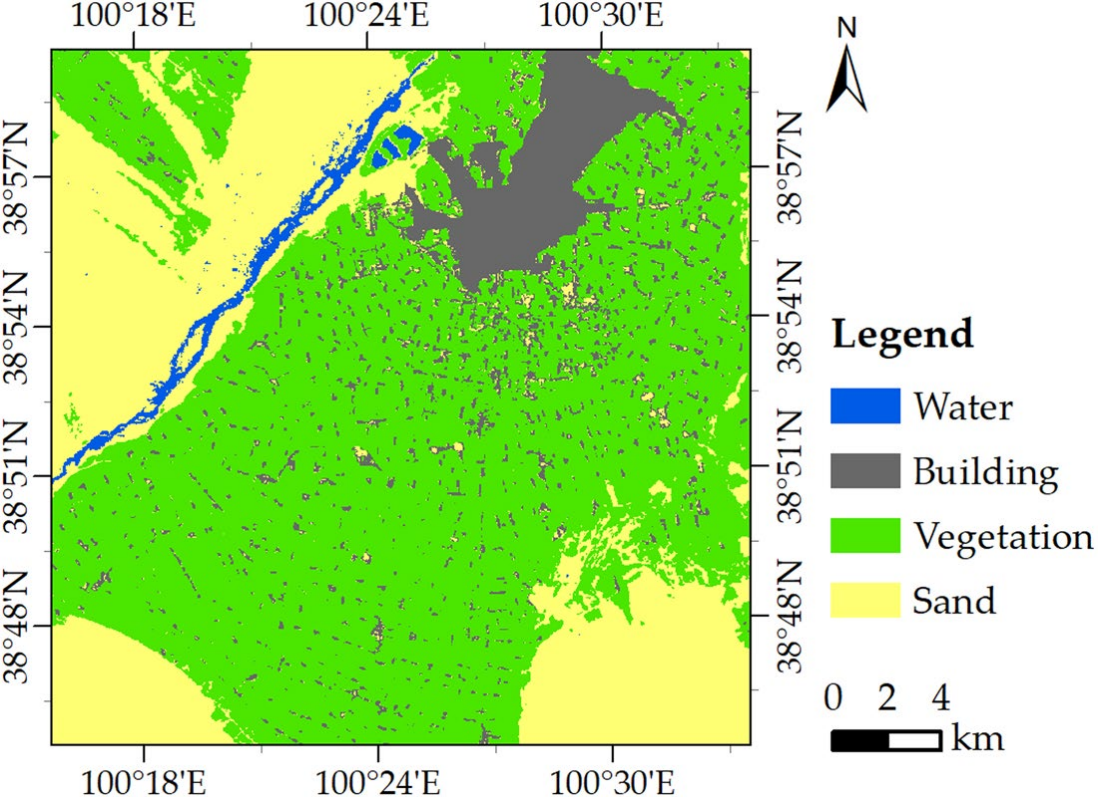
Distribution of land cover in the study area of Zhangye City.

In addition, the land cover dataset was provided by the WestDC. The accuracy and resolution of the land cover dataset were 92.19% and 30 m, respectively^47,48^ (Fig. 14). The accurate land cover dataset is important to present the applicability of our approach in the different land covers.

The ground observations during the time of satellite overpassing were used for validation. The actual LST is estimated from the upwelling and downwelling longwave radiation measured by pyranometers/pyrgeometers using this equation:

9 $${T}_{s}={[\frac{{R}_{lu}-(1-{\varepsilon }_{s})\cdot {R}_{ld}}{{\varepsilon }_{s}\cdot \sigma }]}^{1/4}$$

where *R*_*lu*_ (*R*_*ld*_) is the surface upwelling (downwell) longwave radiation, σ is the Stefan–Boltzmann’s constant.

## Conclusions

The current study improves the original MIRF algorithm by designing a new remote sensing index called NDSI, which can characterize the sandy desert. Owing to NDSI and the independence of land cover datasets, there is an improvement of downscaling LST in the arid region. NDSI can catch the characteristic of the arid desert region, and the distributions of all selected remote sensing indices and downscaled LST match those of the oasis-desert ecosystems. Compared with ground observation, a relatively accurate downscaled LST was generally detected with RMSE, and R^2^ values of 1.25 K and 0.99, respectively. There is a relatively good agreement of the downscaled LST and ground observations at four sites, with bias values of −1.74–1.33 K. In comparison of retrieved 90-m Landsat 8 LST, the R^2^ and RMSE values of downscaled result are 0.97 and 1.62 K, respectively.

In approximately three quarters of the study area, the downscaled LST is reliable. Compared with DisTrad and MIRF approaches, our proposed approach improves the downscaling performance, especially in the arid desert region.

With regard to applicability, our approach worked well (in descending order) in the region of desert, vegetation, water, and impervious surface. Our NDSI-RF approach can obtain the accurate downscaled LST in all seasons for the desert region and in spring and summer for the oasis region. Moreover, our approach can be employed for both the satellite images in the middle-high resolution (e.g., Landsat 8) and LST products in the middle-low resolution (e.g., MODIS). Although our approach is designed for the arid region, such approach also demonstrates the applicability in the humid region and the accuracy of downscaled LST in the humid region, which is not as satisfactory as that in the arid region. Therefore, the applicability of our approach is fully proven for different land covers, seasons, sensors, and climate regions. Moreover, the extensive applicability proves the usefulness of our approach.
